# Avian Influenza Viruses Detected in Birds in Sub-Saharan Africa: A Systematic Review

**DOI:** 10.3390/v12090993

**Published:** 2020-09-07

**Authors:** Annie Kalonda, Ngonda Saasa, Panji Nkhoma, Masahiro Kajihara, Hirofumi Sawa, Ayato Takada, Edgar Simulundu

**Affiliations:** 1 Department of Biomedical Sciences, School of Health Sciences, University of Zambia, Lusaka 10101, Zambia; anniekalonda@gmail.com (A.K.); panjinkhoma@gmail.com (P.N.); 2 Department of Disease Control, School of Veterinary Medicine, University of Zambia, Lusaka 10101, Zambia; nsaasa@gmail.com (N.S.); sawa@czc.hokudai.ac.jp (H.S.); atakada@czc.hokudai.ac.jp (A.T.); 3 Africa Centre of Excellence for Infectious Disease of Humans and Animals, School of Veterinary Medicine, Lusaka 10101, Zambia; 4 Research Center for Zoonosis Control, Hokkaido University, Sapporo 001-0020, Japan; kajihara@czc.hokudai.ac.jp; 5 Global Station for Zoonosis Control, Global Institution for Collaborative Research and Education (GI-CoRE), Hokkaido University Kita-ku, Sapporo 001-0020, Japan; 6 Macha Research Trust, Choma 20100, Zambia

**Keywords:** *Orthomyxoviridae*, avian influenza, avian influenza virus, subtype, ecology, epidemiology, poultry, wild waterfowl, sub-Saharan Africa

## Abstract

In the recent past, sub-Saharan Africa has not escaped the devastating effects of avian influenza virus (AIV) in poultry and wild birds. This systematic review describes the prevalence, spatiotemporal distribution, and virus subtypes detected in domestic and wild birds for the past two decades (2000–2019). We collected data from three electronic databases, PubMed, SpringerLink electronic journals and African Journals Online, using the Preferred Reporting Items for Systematic reviews and Meta-Analyses protocol. A total of 1656 articles were reviewed, from which 68 were selected. An overall prevalence of 3.0% AIV in birds was observed. The prevalence varied between regions and ranged from 1.1% to 7.1%. The Kruskal–Wallis and Wilcoxon signed-rank sum test showed no significant difference in the prevalence of AIV across regions, χ^2^(3) = 5.237, *p* = 0.1553 and seasons, T = 820, z = −1.244, *p* = 0.2136. Nineteen hemagglutinin/neuraminidase subtype combinations were detected during the reviewed period, with southern Africa recording more diverse AIV subtypes than other regions. The most detected subtype was H5N1, followed by H9N2, H5N2, H5N8 and H6N2. Whilst these predominant subtypes were mostly detected in domestic poultry, H1N6, H3N6, H4N6, H4N8, H9N1 and H11N9 were exclusively detected in wild birds. Meanwhile, H5N1, H5N2 and H5N8 were detected in both wild and domestic birds suggesting circulation of these subtypes among wild and domestic birds. Our findings provide critical information on the eco-epidemiology of AIVs that can be used to improve surveillance strategies for the prevention and control of avian influenza in sub-Saharan Africa.

## 1. Introduction

Avian influenza is an acute and highly infectious viral disease caused by influenza A viruses (IAVs) of the genus *Alphainfluenzavirus*, family *Orthomyxoviridae.* Avian influenza viruses (AIVs) are important zoonotic pathogens that may cause high morbidity and mortality resulting in substantial economic losses to the poultry industry [1,2,3]. Migratory avian species within the orders Anseriformes (ducks, geese and swans) and Charadriiformes (gulls, terns and shorebirds) are known to be the natural reservoirs of IAVs [4,5]. While IAVs are principally found in wild aquatic birds, they infect other mammalian species such as humans, horses, pigs, cats, dogs, seals and whales among others [6,7]. Moreover, an IAV-like virus has recently been discovered in bats [8,9], suggesting a possible new natural reservoir host.

IAVs are enveloped, single-stranded, negative-sense RNA viruses with a segmented genome. Eight segments of the IAV genome encode up to 18 proteins: polymerase basic 1 (PB1), polymerase basic 2 (PB2), polymerase acid (PA), hemagglutinin (HA), nucleoprotein (NP), neuraminidase (NA), matrix 1 (M), matrix 2 (M2), nonstructural (NS1), nuclear export protein (NS2/NEP), PB1-F2, PB1-N40, PA-X, M42, PA-N155, PA-182, NS3 and PB2-S1 [10,11,12,13,14,15,16,17,18,19,20,21,22,23,24]. The HA and NA proteins are surface glycoproteins, essential for virus infectivity, and are used in the classification of IAVs into subtypes. Currently, 18 HA and 11 NA subtypes of IAV have been identified, of which 16 HA (H1–H16) and 9 NA (N1–N9) are maintained up to now in avian species [25,26], while 2 HA (H17–H18) and 2 NA (N10–N11) were found in bats [9]. However, some IAV strains have been known to be maintained in mammalian hosts, for example, H1N1 and H3N2 circulate seasonally in humans [27]. Additionally, the HA and NA proteins have the highest evolutionary rates of all influenza virus proteins, which contributes to the genetic and antigenic diversity of these viruses. The genetic diversity is mainly due to two mechanisms, antigenic drift and shift, which involve the accumulation of point mutations over time and the exchange of genome segments between two or more influenza viruses (i.e., genetic reassortment), respectively [28]. These two mechanisms may lead to the emergence of novel strains of IAVs with zoonotic and pandemic potential, which may pose a challenge for control [29].

AIVs can be classified based on their pathogenicity or virulence in chickens as either highly pathogenic avian influenza (HPAI) or low pathogenic avian influenza (LPAI) viruses. The HPAI viruses often become highly pathogenic through the acquisition of multiple basic amino acid residues at the HA cleavage site and are restricted to two subtypes H5 and H7 [30]. However, not all H5 or H7 viruses have the capacity to become HPAI viruses [31,32]. Outbreaks of HPAI viruses constitute a substantial risk to human health, the poultry industry and the global economy [33]. Since the first detection of H5N1 HPAI viruses in Asia, the viruses have spread throughout the world leading to multiple outbreaks affecting millions of birds and considerable human infections [34,35,36]. Moreover, in March 2013, H7N9 LPAI virus emerged in eastern China and caused high morbidity and mortality in humans with an overall case fatality ratio of approximately 37% [37,38]. Furthermore, IAVs have caused at least four major pandemics (1918 “Spanish flu”, 1957 “Asian flu”, 1968 “Hong Kong flu” and the 2009 “swine flu”) in the human population from the 20th century to date, with the worst being the 1918 “Spanish flu” H1N1 pandemic, which recorded nearly 500 million cases and 50 million human deaths globally [39,40]. Although LPAI viruses have typically been known to cause inapparent infections in poultry, some subtypes have caused severe clinical signs in poultry, such as the H9N2 LPAI virus, which has been reported to cause respiratory disease, a reduction in egg production and mortality in birds [41,42].

The importance of poultry farming in sub-Saharan Africa cannot be overemphasized as it is one of the most rapidly growing sectors and an important source of protein and income for several rural households in the region [43]. In Africa, poultry numbers have been estimated to be approximately 1.1 billion [44,45]. Despite the growth of poultry production in the region, the birds are usually reared under poor biosecurity measures, which provide the ideal setting for zoonotic transmission of AIVs [46]. It is also worthy to note that sub-Saharan Africa is a seasonal shelter for a large number of migratory aquatic birds that make their seasonal movements between the temperate zone and the tropics [47], with approximately 5.4 million ducks that gather during the northern winter [48]. These birds congregate and mix with the indigenous water birds in their overwintering sites, which provides opportunities for dissemination and transmission of AIVs between different populations and continents [48].

Despite several studies documenting the presence and impact of AIVs in sub-Saharan Africa [48,49,50,51,52,53,54], there is a lack of consolidated data on the eco-epidemiology of these viruses in birds in the region. Therefore, in this review, we aimed at systematically integrating data from different studies within the region to provide the prevalence, spatiotemporal distribution and virus subtypes detected in domestic and wild birds from January 2000 to December 2019.

## 2. Materials and Methods

### 2.1. Literature Search and Data Collection

To systematically review the literature, we followed the Preferred Reporting Items for Systematic Reviews and Meta-Analyses (PRISMA) protocol 2010 guidelines (S1 Checklist) [55]. Inclusion and exclusion criteria were defined in terms of the relevance of the articles to achieve the study objectives.

A systematic literature search was conducted to identify all publications reporting the detection of avian influenza virus in birds in sub-Saharan Africa between 2000 and 2019. Three electronic databases namely PubMed, SpringerLink electronic journals and African Journals Online (AJOL) were searched using the medical subject headings (MeSH) keywords and Boolean connectors. The following keywords were used: “influenza in birds,” “influenza,” “birds,” “avian,” “avian influenza,” “avian influenza virus,” “sub-Saharan Africa,” “countries in sub-Saharan Africa,” “epidemiology,” “prevalence” and “subtype.” Furthermore, the search was restricted to original articles, titles, abstracts and keywords published in English, which reported on AIVs using serological and/or molecular methods. The last search was conducted on 17th January 2020. All references located in the searches were entered into Endnote, a web-based reference manager. Furthermore, a database was built that included the references of all selected publications, as well as the title, author, year of publication, country or countries where the study was conducted and language of publication. The articles were selected using a two-stage approach. During the first stage, the publications were selected based on their titles and abstracts. During the second stage, the full text of articles selected in the first stage was assessed for eligibility. At this point, the articles that did not meet the inclusion criteria were excluded.

### 2.2. Inclusion Criteria

All study designs were included in this review except experimental studies as these do not represent natural infections. Additionally, studies published between 2000 and 2019, serological, molecular and both serological and molecular studies on AIV in birds in sub-Saharan Africa were investigated. All AIV subtypes and avian species from which the virus was detected were included in this review. Additionally, publications containing data on the positive diagnostic test result, data on incidence, prevalence and distribution of avian influenza in any naturally infected birds were included.

### 2.3. Exclusion Criteria

Studies published before 2000 and after 31st December 2019, editorials, comments/letter to the editor, congress or conference abstracts, review articles, perspectives, personal opinions, theoretical models, pathogenesis models, animal models, case reports in humans, or reports in non-avian species and studies reported in languages other than English were not included. Moreover, studies with the following characteristics were excluded: the diagnostic test not specified, sample source not described, publications reporting data published elsewhere other than sub-Saharan Africa, outbreak reports without laboratory-based confirmation, reporting a zero incidence/prevalence in any diagnostic test, studies with data overlapping with another included study and publications exclusively on the experimental infection. For prevalence and seasonality of AIV analysis, all studies without sampling time, sample size, prevalence or rate were excluded. Additionally, studies with a sample size of less than five were excluded.

### 2.4. Data Extraction

A database on reference information regarding the author’s name, title and year of publication was recorded in the data extraction file. Furthermore, from the included publications, data were extracted on country or countries of study including region, years of sample collection, avian species, the purpose of study, number of samples analyzed, type of samples collected, the diagnostic method(s) used, number of positives and pathogenicity of the AIV subtypes (LPAI or HPAI).

### 2.5. Assessment of Quality and Risk Bias of the Included Studies

To assess the quality and risk of bias of the included studies, the McMaster Critical Review Form—Qualitative Studies (version 2.0) [56] and McMaster Critical Review Form—Quantitative Studies [57] were used.

### 2.6. Statistical Analysis

Data were entered in Microsoft Office Excel 2018 and analyzed using Python 3.7 for Mac. Prevalence and distributions of AIV were reported using descriptive statistics in the form of frequencies, percentages and presented as tables and graphs. Since data were not normally distributed, non-parametric tests such as the Kruskal–Wallis test and Wilcoxon signed-rank sum test were used to determine associations between seasonality, regions and prevalence of AIV. A Pearson correlation was carried out to determine the association between time in years and number of papers published between 2000 and 2019.

## 3. Results

### 3.1. Search Results and Study Selection

**Figure 1:**
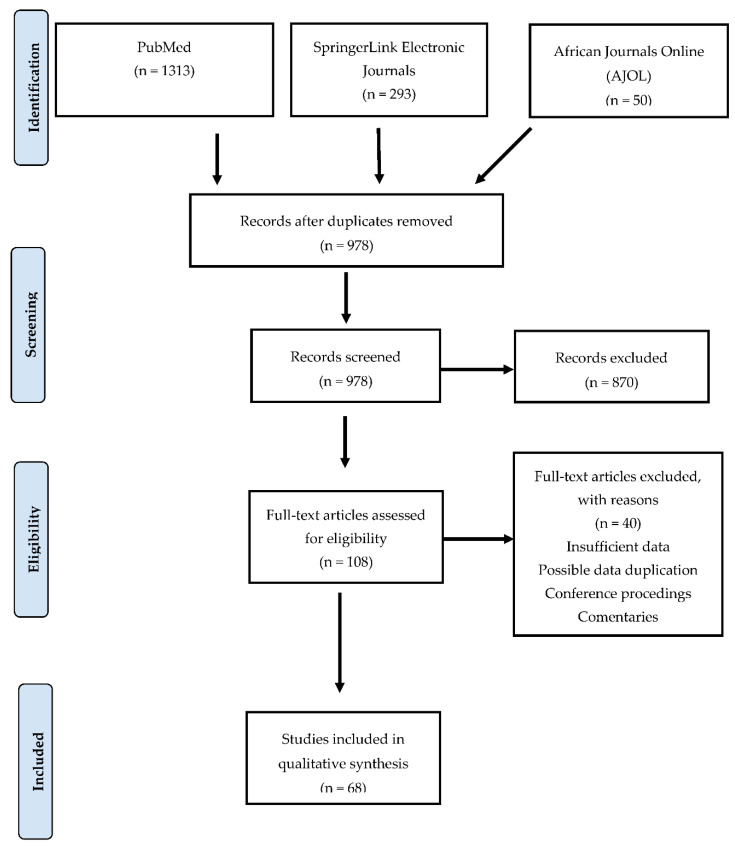
Preferred Reporting Items for Systematic Reviews and Meta-Analyses (PRISMA) flow chart of the literature search, screening, assessing eligibility and article selection.

During the literature search, PubMed yielded 1313 records, SpringerLink electronic journals had 293 and AJOL showed 50 records, giving a total of 1656 research records. Of the 1656 research records, 678 (40.9%) were duplicates and were discarded. Using the set inclusion and exclusion criteria, we screened the remaining 978 records following a flow chart (Figure 1). During the screening, 870/978 (89.0%) articles were excluded based on their titles and abstracts, while 108 articles were retained. The 108 full-text articles were further screened for eligibility and 40/108 (37.0%) were excluded, while a total of 68/108 (63.0%) articles were deemed eligible for inclusion in this systematic review. Table S1 is provided as a Supplemental Material of all included studies.

### 3.2. Characteristics of the Included Studies

**Figure 2:**
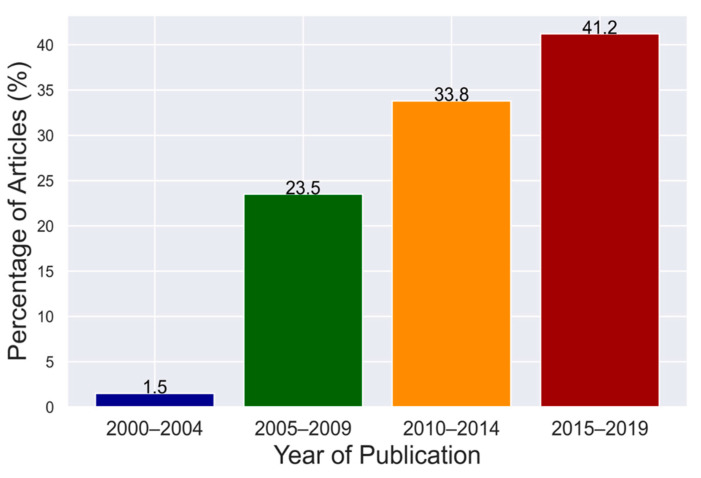
Number of selected articles per quarter from 2000 to 2019.

The reviewed articles were published between 2000 and 2019, from 22 sub-Saharan African countries. One article (1.5%) was published between 2000 and 2004, 16 (23.5%) between 2005 and 2009, 23 (33.8%) between 2010 and 2014 and 28 (41.2%) between 2015 and 2019 (Figure 2).

**Figure 3:**
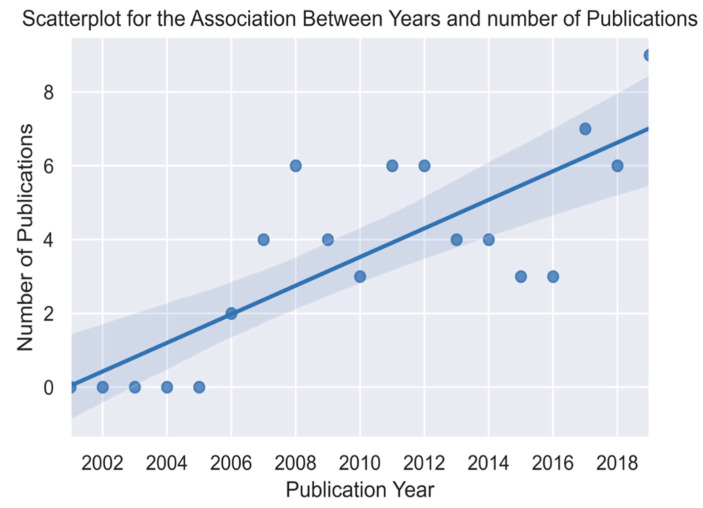
Scatter plot showing the correlation between years and number of publications.

A Pearson correlation was computed to assess the relationship between years of publication and the numbers of articles published. There was a positive correlation between the two variables (r = 0.81, *n* = 20, *p* < 0.0001). This shows that there was a fairly strong positive and significant increase in the number of published articles between the years 2000 to 2019 (Figure 3).

**Figure 4:**
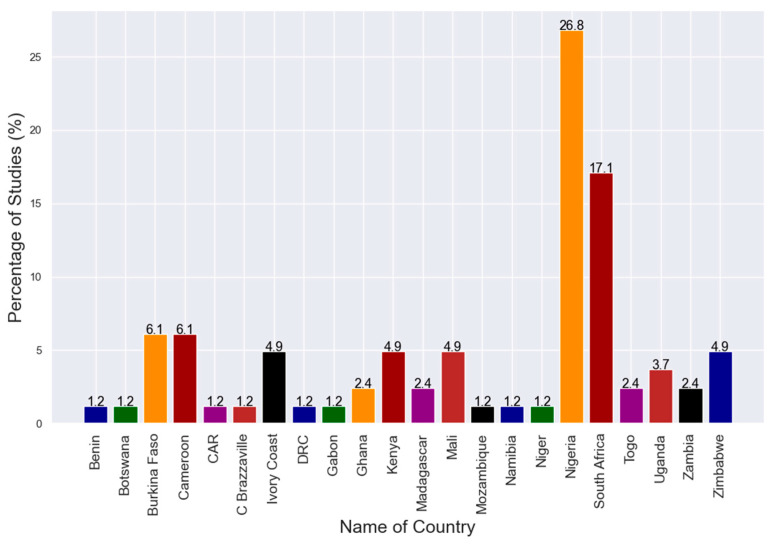
Reviewed articles according to countries.

A review of selected publications yielded a total of 83 published records on the presence of avian influenza in sub-Saharan Africa. Most articles (26.8%) were for studies done in Nigeria followed by those in South Africa (17.1%) (Figure 4).

Of the articles included in this review, 39 (57.4%) were surveillance studies, while 29 (42.6%) were a combination of either case studies or case reports. Additionally, the studies used either molecular, serological or both molecular and serological techniques to detect AIVs in different species of birds. Specifically, 29 (42.6%) studies employed molecular methods, 12 (17.6%) applied serological methods and 27 (39.7%) used both molecular and serological methods.

### 3.3. The Prevalence and Seasonality of AIV in Birds in Sub-Saharan Africa

In many studies, neither prevalence nor sample size was included. Of the 68 studies included in this review, 55 studies with either prevalence values or sample sizes were found. The overall prevalence (determined based on virus isolation and genome detection) and seroprevalence of AIVs and in avian species in sub-Saharan Africa was 3.0% (Table 1) and 4.1% (Table 2), respectively. During the analysis, we divided sub-Saharan Africa into regions namely, Central, East, Southern and West Africa for easier analysis and due to their unique seasons of the year. The prevalence varied between regions and ranged from 1.1% to 7.1% (Table 1), while seroprevalence ranged from 2.2% to 4.1% (Table 2). The majority of the serosurveys were conducted in poultry studies and focused on the detection of H5 and H7 antibodies. We carried out the Kruskal–Wallis test of independent samples and the Wilcoxon signed rank sum test to determine whether the prevalence distribution of AIV was different across seasons and regions in sub-Saharan Africa. Our results showed that there was no significant difference in prevalence across regions χ^2^(3) = 5.237, *p* = 0.1553 and seasons T = 820, z = −1.244, *p* = 0.2136, respectively. However, the highest detection rates of AIVs were observed during the dry season (6.7%) (that is May to October in Central Africa) (Table 3). The lowest prevalence was obtained in the wet season (0.4%) (November to April in Central Africa) (Table 3).

### 3.4. Distribution of the AIV Subtypes and Avian Species

As depicted in Table 4 and Supplementary Table S2, the included studies reported a diverse range of AIV subtypes in different avian species in the past 20 years. According to the analyzed articles, 52/68 (76.5%) of studies specified the AIV subtypes, while 16/68 (23.5%) did not. During the period under review, nine different HA subtypes and six NA subtypes were found in 19 different subtype combinations (Table 4). The 19 AIV subtypes detected were as follows: H1N2, H1N8, H3N6, H3N8, H4N2, H4N6, H4N8, H5N1, H5N2, H5N8, H6N2, H6N8, H7N1, H7N7, H9N1, H9N2, H10N7, H10N9 and H11N9. Southern Africa recorded a wider range of AIV subtype combinations than any other region in sub-Saharan Africa (Table 4). The NA subtypes were not determined for three HA subtypes H5, H6 and H7. Of the 5073 viruses detected, the H5 (78.5%) subtype was the most common, followed by H9 (2.5%), H6 (1.4%), H7 (1.1%) and the rest falling below 1.0%. The H5Nx and H7Nx were either characterized as LPAI viruses or were not characterized at all (Supplementary Table S2). Overall, the most detected subtype combinations were H5N1, followed by H9N2, H5N2, H5N8 and H6N2. The majority of H5N1 and H5N8 subtypes were HPAI viruses and were commonly detected in domestic poultry especially chicken (*Gallus gallus*) and domestic ducks (*Anas platyrhynchos domestica*) (Supplementary Table S2).

**Figure 5:**
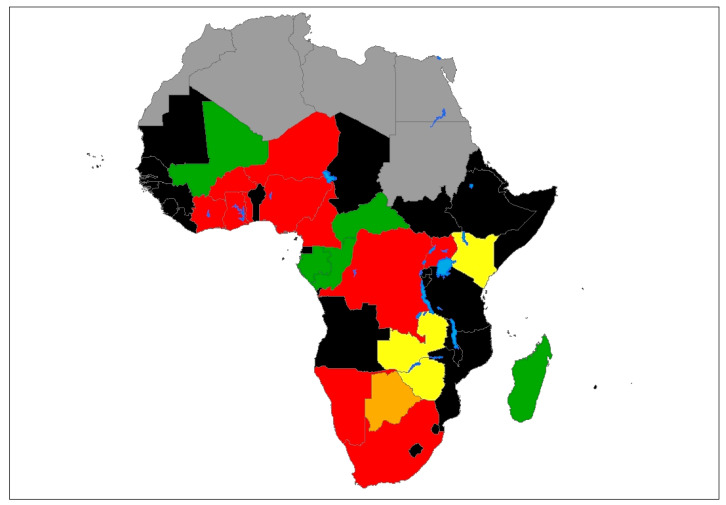
Geographical distribution of highly pathogenic avian influenza (HPAI) and low pathogenic avian influenza (LPAI) subtypes in sub-Saharan Africa. Color codes: red denotes countries reporting either HPAI only or both HPAI and LPAI; orange denotes countries reporting H5 or H7, but whose pathogenicity was not determined; yellow denotes countries reporting LPAI; green denotes countries reporting AIV whose subtypes and pathogenicity were not determined; black denotes countries in sub-Saharan Africa with no reports of AIV in birds in the study period; gray denotes North African countries not included in the study; blue denotes major water bodies.

Overall, the 52 studies that specified the AIV subtypes recorded both HPAI or LPAI viruses among domestic and wild birds (Supplementary Table S2). Majority of the studies that reported HPAIVs were conducted between 2006–2008 and 2017–2018. Furthermore, the majority of studies that reported HPAIVs were conducted in Nigeria 11 (21.2%), followed by Burkina Faso and South Africa 4 (7.6%) each, Cameroon 3 (5.8%) and 2 (3.8%) from Cote d’Ivoire. The Democratic Republic of Congo (DRC), Ghana, Niger, Namibia, Togo and Uganda recorded 1 (1.9%) HPAIV study each. The rest of the studies 22 (42.3%) recorded either LPAIVs or did not specify the pathogenicity of the detected subtype. Zambia and Zimbabwe did not report any HPAIVs according to the 68 studies included in this review (Figure 5). As depicted in Supplementary Table S2, all the HPAIVs from Cameroon, DRC, Ghana, Niger and Togo were only detected in domestic birds, while those from Burkina Faso, Cote d’Ivoire, Nigeria and South Africa were detected in both domestic and wild birds. H5N1, H5N2 and H5N8 HPAI viruses were detected mostly in domestic poultry. Further, the H5N2 HPAI virus was more common among ostriches (*Struthio camelus australis*) than any other bird species included in this study. Notably, the H5N8 HPAI virus was detected in African penguins (*Spheniscus demersus*) in Namibia leading to the most severe mortality on record for this species in Namibia, with more than 350 penguins dead [89]. Furthermore, the H5N1 HPAI virus was detected in Burkina Faso from hooded vultures (*Necrosyrtes monachus*) that exhibited dyspnea and neurological signs [69].

H9N2, H3N8, H5N2, H7N7 and H6N2 LPAI viruses were also detected in domestic poultry. For example, H9N2 LPAI virus detected in Burkina Faso [91] and Ghana [41] and H6N2 LPAI virus in South Africa [92,93] were from symptomatic poultry with signs of respiratory distress, decreased egg production and increased mortality, while in Kenya [64] and Nigeria [78,94] viruses with subtypes H9N2, H7N7, H5N2 and H3N8 were detected in asymptomatic birds. Moreover, mixed infection of H3N8 and H5N2 LPAI viruses was detected in apparently health turkeys (*Meleagris* Spp.) in Nigeria. Furthermore, H1N6, H3N6, H4N6, H4N8, H9N1 and H11N9 LPAI viruses were exclusively detected in wild birds.

### 3.5. Distribution of AIVs among Different Avian Species

The distribution of AIVs among different avian species according to the articles included in this review is depicted in Table 5. The majority of the studies were conducted in domestic birds with the highest number of studies (35) being in chicken (*Gallus gallus*) and (20) in domestic ducks (*Anas platyrhynchos domestica*) in 12 and 10 different countries, respectively (Table 5). Notably, studies in ostriches were reported only in South Africa [83,86,95,96,97,98,99]. In wild birds, most studies (6) were in Egyptian geese (*Alopochen aegyptiacus*) done in Kenya, South Africa and Zambia followed by 4 studies in unspecified wild birds. For the rest of the free-flying and aquatic birds, 1 to 3 studies were conducted in various sub-Saharan African countries as shown in Table 5.

## 4. Discussion

This systematic review is aimed at investigating the prevalence, spatiotemporal distribution, and virus subtypes of AIVs detected in domestic and wild birds over a two-decade period (2000–2019) in sub-Saharan Africa. We retrieved many study articles from the databases, but only 68 studies from 22 different countries in sub-Saharan Africa met our inclusion criteria. Our findings showed a significant increase in the number of studies published between 2000 and 2019. This increase could be attributed to the increase in the number of AIV outbreaks recorded within the region, with Nigeria recording 1205 suspected outbreaks [100] between 2006 and 2007. Additionally, there has been an increase in surveillance studies [49,51,66,67,85,98,101,102,103,104] regionally and in different countries, improved laboratory diagnostic capacity and generally enhanced surveillance systems aimed at preventing HPAI outbreaks.

The AIV prevalence generally varied from region to region with the highest prevalence being reported in Central Africa (7.1%) and the lowest in East Africa (1.1%) (Table 1). Moreover, our findings indicate that AIVs were detected throughout the year in sub-Saharan Africa with higher prevalence during the dry season. Whilst it is generally understood that the prevalence of AIV infection tends to increase during the period when Eurasian migratory water birds overwinter in sub-Saharan Africa and decrease after they migrate back to Eurasia [105], this study revealed a higher prevalence in the dry season when Eurasian migratory birds are absent or rare. It is possible that the limited water bodies in the dry season may allow increased interaction of waterfowl by congregating at particular sites, which provides opportunities for AIV transmission as well as detection during surveillance activities. However, despite the observed variations in prevalence and seasonality of AIV infection, the difference was not statistically significant. Further, the finding indicates that AIVs are perpetuated in migratory water birds originating from Eurasia as well as in indigenous African species that remain in the continent throughout the year.

Wild migratory aquatic birds are known to be the natural reservoir of AIVs globally [4,5]. Therefore, it is not surprising that different wild birds in sub-Saharan Africa harbor AIVs according to the findings of this review. Our findings also highlight the impact of AIVs on migratory and non-migratory local birds. Analysis of HA subtype diversity revealed that H5 was the most predominant HA subtype detected in this review followed by H9, H6 and H7. The predominance of these subtypes could be attributed to the fact that most of the AIV surveillance activities have concentrated on the detection of subtypes H5, H7 and/or H9 [106,107] and that they cause dramatic devastation, particularly HPAI that is difficult to miss. The H5 and H7 viruses were detected in both wild and domestic birds implying possible transmission from wild birds to domestic birds or vice versa.

The HA subtype diversity in our study has similarities and differences to that found in studies in China [107], Netherlands [108], North America [109,110], Germany [111] and Northern Europe [112] though a number of these studies were done in wild aquatic birds. For example, our study was comparable to the study in China [107] and that in the Netherlands [108], which did not detect H8, H12–H16. Furthermore, our study only detected six NA subtypes and 19 HA/NA combinations, while studies in Germany [111], Northern Europe [112] and North America [109,110] detected 40 or more subtype combinations. This higher subtype diversity in these studies could be because the surveillance was focused on wild waterfowl, which are expected to harbor a large pool of various AIV subtypes, which could also be true for Southern Africa, which had the highest subtype diversity in sub-Saharan Africa. The most prevalent NA subtype was N1, followed by N2 and N8, while N3–N5 and H2, H8 and H12–H16 were never detected, which may be due to limited surveillance efforts in wild birds or that there may be avian species-specific niches of certain HA and NA subtypes in the studied region.

Furthermore, the findings of this study revealed the presence of LPAI and HPAI viruses in both wild and domestic birds. However, the presence of HPAI viruses was more common among domestic birds, with the highest detection rate being in chickens and ducks. The dominant subtype was H5N1, which circulated in both wild and domestic birds. Our results further demonstrated that West African countries were the most hit by the H5N1 HPAI viruses with at least Nigeria [100,113], Niger [81], Ghana [81], Burkina Faso [69,113], Cote d’Ivoire [68] and Togo [71] recording one or more outbreaks during the evaluated period. This may be because West Africa is a major wintering area for migratory water birds (Anseriformes and Charadriiformes), which are the natural reservoirs of AIVs [5]. Moreover, the H5N1 HPAI viruses have persisted within the West African ecosystem since their introduction in Nigeria in 2006 [35,114]. The persistence and transmission of H5N1 HPAI viruses in West Africa have been attributed to the illegal movement of infected poultry and products, multispecies livestock farming and poor biosecurity compliance levels in live bird markets (LBMs) [35,115]. Furthermore, an HPAI outbreak caused by H5N1 viruses has been reported in Cameroon, a Central African country that shares borders with Nigeria, which suggests transboundary transmission due to porous borders, leading to illegal trade in livestock, especially birds, between these countries [58].

Apart from the H5N1 HPAI viruses, the study also revealed the presence of other HPAI virus subtypes namely H5N8 and H5N2 in different avian species in sub-Saharan Africa. The first case of H5N8 HPAI infection in Africa was reported around the same time in Egypt and Nigeria and later spread to other neighboring countries [52]. This outbreak spread to Uganda, South Africa, Zimbabwe and DRC [52,53,63,116,117]. Additionally, HPAI outbreaks caused by H5N2 viruses have been reported in farmed ostriches in South Africa. These outbreaks caused a devastating impact on the ostrich industry of South Africa, which account for at least 65% of global ostrich production [118]. The presence and spread of H5N8 and H5N2 HPAI viruses have been attributed to migratory waterfowl due to the long-distance seasonal movements along their migration routes and also the other sedentary birds that have been implicated in facilitating the intracontinental dissemination of the virus [117]. In fact, H5N2 HPAI viruses have been detected in apparently healthy wild waterfowl in Nigeria [72]. Although HPAI viruses have been detected in many countries in sub-Saharan Africa, our results did not report any HPAI viruses in Zambia and Zimbabwe. Further, other countries such as Mali, Central Africa Republic, Congo-Brazzaville, Gabon and Madagascar did not specify the subtypes and pathogenicity of the viruses detected. However, the presence of HPAI viruses highlights the importance of continued and better epidemiological monitoring systems to allow their timely detection for mitigatory measures.

A large diversity of LPAI virus subtypes was detected in this review with H9N2 being the most predominant followed by H6N2 and H3N8. The H9N2 and H6N2 LPAI viruses were exclusively detected in domestic birds, in which they caused asymptomatic or symptomatic infections. Symptomatic infections caused by LPAI viruses include severe clinical signs in poultry, such as respiratory distress, intestinal signs and a drop in egg production [42]. These observations are consistent with previous studies in Iraq [119]. Moreover, H9N2 AIV infection is known to be endemic among poultry in Eurasia [120,121], and its circulation has been reported in North Africa, Europe and Asia among others [42,120,121,122,123,124]. H9N2 viruses are also known to circulate between wild birds and poultry sold at LBMs. LBMs are known to be reservoirs, amplifiers and sources of AIVs [46]. Furthermore, the transmission of H9N2 viruses from poultry to humans has been reported [125].

While H4N6 and H7N7 were the most prevalent AIV subtypes detected in Northern Europe and Germany [111,112], these subtypes were among the least detected in this review. H3N6 and H9N1 were the least and the only AIV subtypes detected in the great white pelican (*Pelecanus onocrotalus*) in Zambia [49,51]. Further, the presence of AIVs in wild waterfowl such as white-winged black terns (*Chlidonias leucopterus*), Egyptian geese (*Alopochen aegyptiacus*), yellow-billed duck (*Anas undulata*), shelduck (*Tadoma cana*) among others is important as these birds are known to be the primary reservoir of AIVs. Although our data seem to suggest an increase in the incidence of AIV infection in migratory waterfowl and domestic birds, this review also reports the detection of H5N1 HPAI viruses in African wild birds, hooded vultures (*Necrosyrtes monachus)* in Burkina Faso [69]. The detection of AIVs in wild migratory birds and minor bird reservoirs highlights the important role they play in the maintenance and transmission of these viruses. AIVs with H5Nx, H7Nx and other subtypes (not fully identified) were detected in Afro-tropical waterfowl and swallows in Zimbabwe throughout the year, and the detection rate was higher when Palearctic birds were present, suggesting the yearly persistence of LPAI viruses in Afro-tropical waterfowl and other wild birds [126].

Although the benefits of systematic reviews are enormous, they are also not short of the challenges and limitations that come with aggregating data. For example, the distribution of AIV subtypes and avian species might not be limited to the present findings since our study included only publications with original data, well-elaborated methodological approach and laboratory-confirmed cases of AIVs. Furthermore, we only searched three recognized electronic databases and only included articles written in English, making it possible to leave out studies or publications that may be relevant to this review. Avian influenza subtypes depicted in this study may not be the true reflection of the subtype diversity in sub-Saharan Africa as some studies did not perform subtype identification due to insufficient samples and lack of laboratory capacity for influenza diagnostics.

## 5. Conclusions

Our review of AIVs in sub-Saharan Africa has provided an insight into the ecology and epidemiological trends of AIVs in birds over a twenty-year period (2000–2019). We found a considerable diversity of AIV subtypes in sub-Saharan Africa, with some subtypes being detected frequently in both wild and domestic birds. Furthermore, AIV was detected in wildfowl and domestic birds in both the wet and dry seasons, with viruses being detected in both Eurasian migratory and indigenous African wild birds. These results suggest a year-round perpetuation of AIVs in Afrotropical ecosystems, with seasonal variation. Continued surveillance, especially in wild birds, to better understand the eco-epidemiology of IAVs, along with improved biosecurity on poultry farms, enhanced extension services and engagement of various disciplines under a “One Health” approach in tackling avian influenza could assist in mitigating the impact of AIV in sub-Saharan Africa.

## Figures and Tables

**Table 1:** Prevalence of avian influenza virus (AIV) in sub-Saharan Africa.

Study Region	No. of Studies	No. Samples	No. Positive	Prevalence (%)
Central Africa ^a^	4	4868	344	7.1
East Africa ^b^	6	13,875	146	1.1
West Africa ^c^	19	38,203	1269	3.3
Southern Africa ^d^	12	12,518	349	2.8
Total	41	69,464	2108	Overall prevalence: 3.0

Study regions: ^a^ Central African Republic, Congo Brazzaville, Cameroon and Democratic Republic of Congo, ^b^ Kenya, Uganda, ^c^ Benin, Burkina Faso, Ivory Coast, Gabon, Ghana, Mali, Niger, Nigeria, Togo, ^d^ Botswana, Namibia, South Africa, Zambia, Zimbabwe.

**Table 2:** Seroprevalence of AIV in sub-Saharan Africa.

Study Regions	No. of Studies	No. of Samples	No. Samples	Seroprevalence (%)
Central Africa ^a^	0	-	-	-
East Africa ^b^	2	3517	77	2.2
West Africa ^c^	11	16,669	875	5.2
Southern Africa ^d^	4	235,084	9605	4.1
Total	18	255,270	10,557	Overall seroprevalence: 4.1

Study regions: ^a^ Central African Republic, Congo Brazzaville, Cameroon and Democratic Republic of Congo, ^b^ Kenya, Uganda, ^c^ Benin, Burkina Faso, Ivory Coast, Gabon, Ghana, Mali, Niger, Nigeria, Togo, ^d^ Botswana, Namibia, South Africa, Zambia, Zimbabwe.

**Table 3:** Prevalence of AIV according to regions and seasons ^a^.

Study Region	No. of Studies	No. of Samples	Dry Season (%)	Wet Season (%)	Reference
**Central Africa**	4	4868	325 (6.7%)	19 (0.4%)	[53,58,59,60]
**East Africa**	5	9556	71 (0.7%)	46 (0.5%)	[45,61,62,63,64]
**West Africa**	18	11,680	319 (2.7%)	158 (1.4%)	[41,65,66,67,68,69,70,71,72,73,74,75,76,77,78,79,80,81]
**Southern Africa**	11	6009	165 (2.7%)	48 (0.7%)	[49,51,82,83,84,85,86,87,88,89,90]
**Total (Overall Prevalence%)**	38	32,113	880 (2.7%)	271 (0.8%)	

^a^ In sub-Saharan Africa, seasons differ according to the regions: Central/Southern Africa—dry season (May–October) and wet season (November–April); East Africa—dry season (January–March/June–October) and wet season (April–June/November–December); West Africa—dry season (January–March/June–October) and wet season (April–June/November–December).

**Table 4:** AIV subtype detection in sub-Saharan Africa according to the included studies.

Study Region	Central Africa	East Africa	Southern Africa	West Africa
HA Subtypes Detected	H5	H4, H5, H9	H1, H3, H4, H5, H6, H7, H9, H10, H11	H3, H5, H7, H9
Most Prevalent HA Subtypes	H5	H5	H5	H5
HA Subtypes not Detected	H1–H4, H6–H16	H1–H3, H6–H8, H10–H16	H2, H8, H12–H16	H1–H2, H4, H6, H8, H10–H16
Prevalent NA Subtypes	N1, N8	N2, N6, N8	N1, N2, N6, N7, N8, N9	N1, N2, N7, N8
NA Subtypes not Detected	N2, N3, N4, N5, N6, N7, N9	N1, N3, N4, N5, N7, N9	N3, N4, N5	N3, N4, N5, N6, N9
Prevalent Subtype Combinations	H5N1, H5N8, H5Nx	H4N6, H5N8, H9N2, H5Nx	H1N2, H1N8, H3N6, H3N8, H4N2, H4N6, H4N8, H5N1, H5N2, H5N8, H6N2, H6N8, H9N2, H10N7, H10N9, H11N9, H5Nx, H6Nx, H7Nx	H3N8, H5N1, H5N2, H7N7, H9N2, H5Nx, H7Nx
Most Prevalent Subtype Combinations	H5N1	H5N8	H5N2, H5N8, H6N2	H5N1, H9N2

Note: HA = hemagglutinin; NA = neuraminidase; Nx = unknown NA subtype.

**Table 5:** Avian host range of AIV according to the 68 publications included in the review.

Host	Number of Publications *	Number of Countries	Names of Countries
**Domestic Birds**			
Chicken	35	12	Burkina Faso, Cameroon, Cote d’Ivoire, Ghana, Kenya, Madagascar, Mali, Nigeria, South Africa, Togo, Uganda
Domestic duck	20	10	Cameroon, Cote d’Ivoire, DRC, Ghana, Kenya, Madagascar, Mali, Nigeria, South Africa, Uganda
Ostrich	8	1	South Africa
Turkey	7	7	Cameroon, Cote d’Ivoire, Kenya, Mali, Nigeria, South Africa, Uganda
Domestic guinea fowl	7	5	Burkina Faso, Cameroon, Mali, Nigeria, South Africa
Pigeon	5	3	Cameroon, Nigeria, South Africa
Domestic geese	4	4	Cameroon, Kenya, Madagascar, South Africa
Poultry ^a^	3	3	Nigeria
Indian peafowl	2	1	Cameroon
**Wild Birds**			
Egyptian goose	6	3	Kenya, South Africa, Zambia
Wild species ^a^	4	3	Nigeria, Africa, South Africa
White-faced whistling duck	3	3	Kenya, Nigeria, Mali
Yellow-billed duck	3	2	Kenya, South Africa
Hooded vulture	3	1	Burkina Faso
Red-billed quelea	2	1	Mali, Zimbabwe
Red-billed teal	2	1	South Africa
Great white pelican	2	1	Zambia
Spur-winged goose	2	1	Nigeria
Duck	2	2	Zambia, Zimbabwe
Cattle egret	2	2	Nigeria, Zimbabwe
Barn swallow	2	1	Zimbabwe
African sacred ibis	2	1	South Africa
Turtle dove	2	1	Cote d’Ivoire
Cape teal	2	2	Kenya, South Africa
African penguin ^b^	1	1	Namibia
Sparrowhawk	1	1	Cote d’Ivoire
Dove	1	1	Cote d’Ivoire
Crow	1	1	Cote d’Ivoire
Weaver	1	1	Cote d’Ivoire
Hottentot teal	1	1	Kenya
Red-knobbed coot	1	1	Kenya
Garganey	1	1	Mali
Ruff	1	1	Mali
Northern pintail	1	1	Mali
Purple swamphen	1	1	Mali
Common moorhen	1	1	Mali
Comb duck	1	1	Mali
Gull-billed tern	1	1	Mali
Spotted redshank	1	1	Mali
Speckled pigeon	1	1	Nigeria
Canada goose	1	1	Nigeria
Gray crown crane	1	1	Nigeria
African gray parrot	1	1	Nigeria
Cape shoveler	1	1	South Africa
Swift tern	1	1	South Africa
White-winged black tern	1	1	South Africa
Hadada ibis	1	1	South Africa
Shelduck	1	1	South Africa
Brown-throated martin	1	1	Kenya

* Multiple publications reported on multiple animal species; ^a^ poultry and wild species not clearly specified; ^b^ sea bird; wild birds include free-flying wild birds and aquatic waterfowl.

## Supplementary Materials

The following are available online at https://www.mdpi.com/1999-4915/12/9/993/s1, S1 Checklist-PRISMA checklist. Table S1. References of all included studies. Table S2. Countries with reported AIV of diverse subtypes and avian species.
